# Correspondence to editorial “Oral health in southeast Asia: addressing inaccessibility”

**DOI:** 10.1016/j.lansea.2025.100550

**Published:** 2025-02-21

**Authors:** Romain Lan, Laurie Fraticelli, Denis Bourgeois, Florence Carrouel

**Affiliations:** a Laboratory Health Systemic Process (P2S), UR4129, University Claude Bernard Lyon 1, University of Lyon, Lyon, France; b Laboratory ADES, Aix Marseille University, CNRS, EFS, Marseille, France

As the last editorial points out,^1^ unequal distribution of services and lack of effective prevention policies in southeast Asia exacerbate health inequities, affecting vulnerable populations such as rural communities and low-income groups.

This challenge is exacerbated by limited access to trained professionals, and insufficient oral health education at both individual and professional levels. The absence of structured personalized prophylaxis protocols weakens preventive measures, contributing to the high prevalence and incidence of oral diseases.^2^

First, these diseases are often linked with oral microbiome dysbiosis, leading to systemic consequences, particularly in aging populations and individuals with cancer or chronic conditions. Growing evidence on oral-systemic interactions underscores the need to integrate oral health into comprehensive disease management.^3^

Second, oral cancer remains a major public health challenge in Southeast Asia, where betel quid chewing significantly increases cancer risk.^4^ The high prevalence of smokeless tobacco use, combined with cultural and socioeconomic factors, exacerbates the disease burden. Given its severe health and economic impact, urgent measures are needed.^5^

To address this issue, a comprehensive and integrated approach to oral health is essential, combining preventive and therapeutic strategies. Key measures should involve systematic screening for high-risk individuals, early oral hygiene education, enhanced training for healthcare providers, and community-based health promotion. Additionally, integrating digital solutions—along with strengthening primary healthcare and fostering a supportive policy environment—can improve accessibility and efficiency. Equally crucial are public awareness campaigns to curb tobacco and betel quid consumption, reducing oral cancer's toll.

## Contributors

Conceptualization: Romain Lan; Writing original draft: Romain Lan and Florence Carrouel; Writing review and editing: Romain Lan, Laurie Fraticelli, Denis Bourgeois and Florence Carrouel.

## Declaration of interests

None.
